# The Fusiform Face Area Is Engaged in Holistic, Not Parts-Based, Representation of Faces

**DOI:** 10.1371/journal.pone.0040390

**Published:** 2012-07-06

**Authors:** Jiedong Zhang, Xiaobai Li, Yiying Song, Jia Liu

**Affiliations:** 1 State Key Laboratory of Cognitive Neuroscience and Learning and IDG/McGovern Institute for Brain Research, Beijing Normal University, Beijing, China; 2 Graduate University of Chinese Academy of Sciences, Beijing, China; National Institute of Mental Health, United States of America

## Abstract

Numerous studies with functional magnetic resonance imaging have shown that the fusiform face area (FFA) in the human brain plays a key role in face perception. Recent studies have found that both the featural information of faces (e.g., eyes, nose, and mouth) and the configural information of faces (i.e., spatial relation among features) are encoded in the FFA. However, little is known about whether the featural information is encoded independent of or combined with the configural information in the FFA. Here we used multi-voxel pattern analysis to examine holistic representation of faces in the FFA by correlating spatial patterns of activation with behavioral performance in discriminating face parts with face configurations either present or absent. Behaviorally, the absence of face configurations (versus presence) impaired discrimination of face parts, suggesting a holistic representation in the brain. Neurally, spatial patterns of activation in the FFA were more similar among correct than incorrect trials only when face parts were presented in a veridical face configuration. In contrast, spatial patterns of activation in the occipital face area, as well as the object-selective lateral occipital complex, were more similar among correct than incorrect trials regardless of the presence of veridical face configurations. This finding suggests that in the FFA faces are represented not on the basis of individual parts but in terms of the whole that emerges from the parts.

## Introduction

Studies with functional magnetic resonance imaging (fMRI) have identified a face-selective region in the fusiform gyrus, termed the fusiform face area (FFA) [1]. Further studies examined the nature of representations extracted from faces in the FFA, and found that both the featural information of faces (e.g., eyes, nose, and mouth) and the configural information of faces (i.e., the spatial relation among face parts) are encoded in the FFA [2]–[9]. However, whether the featural information is encoded independent of or combined with the configural information in the FFA is less clear. Here we used fMRI to examine the representation of faces in the FFA while manipulating the configural information.

Extensive behavioral studies have shown that the key difference in the way that faces are processed, compared to non-face objects, is that the featural and configural information are processed together as an integrated whole, termed holistic processing [10]–[14]. Further fMRI studies have suggested that the FFA is a neural substrate for holistic representation of faces. For example, the neural response to the featural information was found to be correlated with that of the configural information in the FFA [5], and configural changes affected FFA responses to face parts [15], [16]. On the other hand, the processing of the featural information alone, termed parts-based processing, also plays an important role in recognizing faces [8], [17]. Similarly, the FFA shows no preference for the processing of the configural information over the featural information [3], [4], [9], suggesting that the FFA may be engaged in the parts-based representation of faces as well.

**Figure 1:**
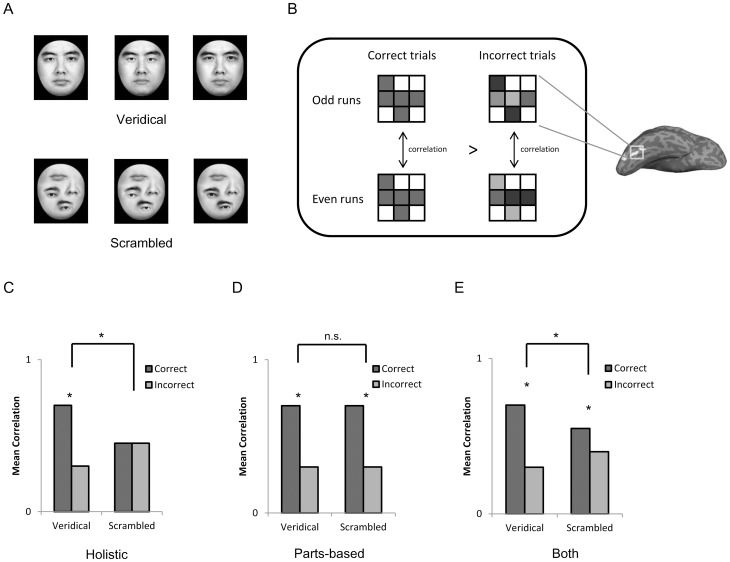
Stimuli and hypothetical representations. *A*) Exemplars of the Veridical face set (top) and the Scrambled face set (bottom). Face stimuli differ either in eyes or mouths within each set. *B*) Illustration of behavior-neural activation correlational analysis. Correlations of spatial patterns of activation in the FFA were calculated across independent fMRI data sets (i.e., even versus odd runs). We then examined whether the correlation of spatial patterns was higher for correct than incorrect trials. *C*) “Holistic” representation. If the featural information of faces is combined with the configural information, a higher correlation of spatial patterns in correct trials than incorrect trials shall be observed only when the veridical face configuration is present (i.e., the interaction). *D*) “Parts-based” representation. If the featural information of faces is encoded independently of the configural information, a higher correlation of spatial patterns in correct trials than incorrect trials shall be observed regardless of whether or not the veridical face configuration is presented (i.e., the main effect). *E*) “Both” representation. If both types of representations are implemented in the FFA, we shall expect both the interaction and the main effect.

Here we directly tested whether the representation of faces in the FFA is holistic, parts-based, or both. To do this, we examined the effect of configural change on the representation of the featural information in the FFA. Studies of facial structure have drawn two distinctions on configural information: (1) first-order configuration (i.e., the T-shaped configuration of eyes above nose above mouth) and (2) second-order configuration (i.e., the precise metrical relationships between face parts) [18], [19]. Similar to holistic processing, the processing of the second-order configuration, termed configural processing, is critical in determining the identity of an individual face [20]–[24]. However, holistic processing and configural processing are two separate and distinct processes [10]. Therefore, to examine whether faces are holistically represented in the FFA, we asked the more basic first-order question of how the representation of faces is affected by the mere presence (vs. absence) of the first-order configuration. Specifically, we used a variant of the whole-part task, where participants are better at discriminating face parts (e.g., the eyes) between two serially-presented faces when the first-order configuration is present (Fig. 1*A*, Veridical) than absent (Fig. 1*A*, Scrambled) [5], [13], [20], [25], [26]. The performance difference in discriminating face parts between the presence and absence of the first-order face configuration was used as an index of holistic processing [13], [27], [28].

To examine neural correlates of behavioral holistic processing, we used multi-voxel pattern analysis (MVPA) to measure neural information encoded in the FFA. As compared with the traditional measure of the mean magnitude of blood oxygen level-dependent (BOLD) responses pooled across voxels, voxel-by-voxel patterns of activation provide richer information on neural representations at a finer-scale [29]–[33]. Further, we examined whether the spatial pattern of activation in the FFA is behaviorally relevant or simply epiphenomenal by correlating neural activation with behavioral measures of face perception. Previous studies have shown that greater similarity of spatial patterns was associated with conscious (versus unconscious) experiences [34] and better behavioral performance in memory retrieval [35]. It is then proposed that greater similarity of spatial patterns may reflect the fact that neuronal coding is more stable [34] and more effective [35]. Accordingly, it is likely that greater similarity of neural patterns is related to better behavioral performance in face perception. Specifically, we calculated correlations of spatial patterns of activation across independent fMRI data sets separately for correct versus incorrect trials (Fig. 1B). If spatial patterns of activation in a region are behaviorally relevant, correlations of spatial patterns shall be higher for correct than incorrect trials [35]–[38].

Based on these designs, we predicted that: (1) if the FFA is engaged in holistic representation of faces (Fig. 1*C*), we shall see an interaction, with a higher correlation occurring when participants correctly discriminate face parts placed in a veridical face configuration, not in a scrambled face configuration; or (2) if the FFA is engaged in the parts-based representation of faces (Fig. 1*D*), we shall see a main effect of higher correlations of spatial patterns for correct than incorrect trials regardless of configural changes; or (3) if the FFA is engaged in both types of representation (Fig. 1*E*), we should observe both the interaction and the main effect.

## Materials and Methods

### Participants

Thirteen college students (age 20–30; 8 females) participated in the study. All participants were right-handed and had normal or corrected-to-normal visual acuity. The fMRI protocol was approved by the IRB of Beijing Normal University, Beijing, China. A written consent form was collected before the experiment from each participant.

### Stimuli

The face stimuli were gray-scale adult Chinese faces with external contour (a roughly oval shape with hair on the top and sides) removed. Three original male faces were used to generate the stimuli and all stimuli were 7×8.3 cm (5.7°×6.8° visual degrees in the scanner). First, a face template containing eyebrows and a nose was created. Second, for the face set with a veridical face configuration (Veridical), the eyes and mouth of a face image were randomly selected from those of three original faces, and then placed in a veridical face configuration. The metrical relationship between face parts (i.e., the second-order configuration) of all face exemplars was kept as constant as possible (Fig. 1*A*, top). Third, to create the face set with a non-face configuration (Scrambled), face parts from the Veridical face set were moved to new locations [5], [20], [25] (Fig. 1*A*, bottom). This non-face configuration was kept constant for all face images in the set. Therefore, participants could expect the left eye at one position and the nose at another, just as they could for faces with a veridical configuration. Note that in this study we only tested the effect of configural changes on the perception of face parts. The effect of featural changes on the perception of face configurations was not examined simply because changes in featural information inevitably produce concurrent changes in configuration [22], [39], [40].

### Experimental design

Each participant finished a single session consisting of (1) two blocked-design functional localizer runs, and (2) ten event-related-design experimental runs. The localizer runs consisted of human frontal-view faces, objects, houses, and scrambled objects. Each localizer run lasted 5 min and 36 sec, and consisted of sixteen 16 sec blocks with five 16 sec fixation periods interleaved. During each block, twenty different exemplars of a given stimulus category were presented for 300 msec in the center of the screen, followed by a blank interval of 500 msec. During the localizer runs, participants pressed a button whenever they saw two identical images in a row (i.e., 1-back task).

**Figure 2:**
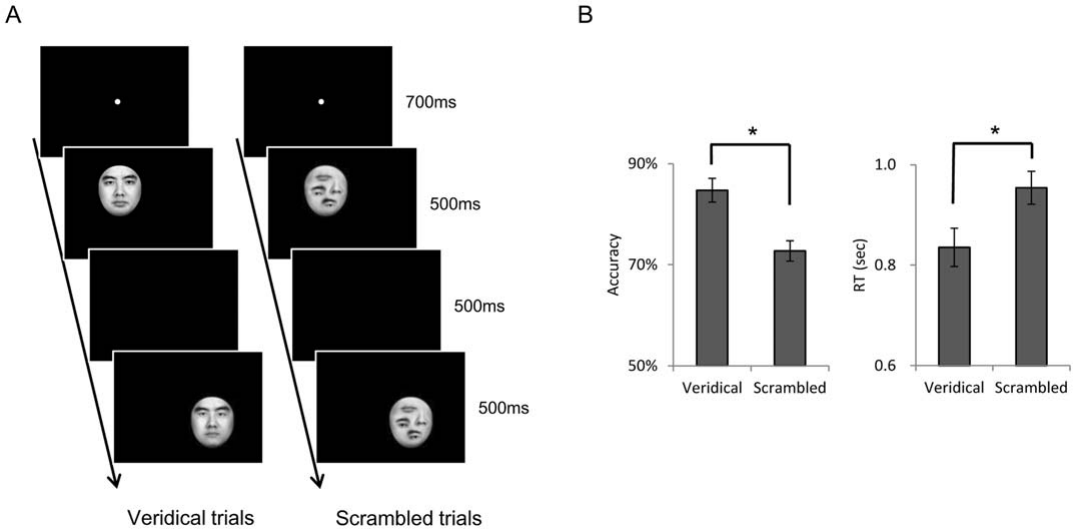
Experimental procedure and behavioral result. *A*) Sample trials in the experimental runs. Participants were instructed to discriminate face parts in a sequentially-presented face pair, either both with veridical face configurations (Veridical trials) or both without the configurations (Scrambled trials). The two types of trials were randomly intermixed in the runs. *B*) Behavioral performance. In the scanner, participants were more accurate (Left) and quicker (Right) in discriminating face parts in the Veridical trials than the Scrambled trials. Error bars indicate standard error of mean (s.e.m.) above and below the mean. *: *p*<0.001.

During the experimental runs, participants performed a discrimination task on pairs of faces that could differ in face parts. In a trial, a pair of face images was presented sequentially, either both with or without a veridical face configuration (Fig. 2*A*). Each trial lasted 4 sec, starting with a blank screen of 700 msec, followed by the first face image presented in the upper-left quadrant of the screen for 500 msec. Then, after a blank interval of 500 msec, the second image was presented in the lower-right quadrant for 500 msec, followed by a blank screen of 1.8 sec. The displacement of the first and second images in the screen was to discourage the use of low-level visual information for performing the task. In each run, there were 36 trials for the Veridical condition and Scrambled condition respectively, half of which consisted of face pairs that were identical and half of which consisted of face pairs that differed in either eyes or mouths. Participants were instructed to discriminate face parts between the face pair and then made a “same” or “different” judgment upon seeing the second image. There were a total of 360 trials for each condition in the experimental runs. In addition, twelve 2 sec fixation trials (i.e., no stimulus presented) were included as temporal jitters in each run. The order of conditions was counterbalanced using the optseq2 program (http://surfer.nmr.mgh.harvard.edu/optseq/) so that trials from each condition (including the fixation trials) were preceded, on average, equally often by trials from each of the other conditions. In addition, there was an 8 sec fixation period at the beginning and at the end of each run. Thus, each experimental run lasted 5 min and 28 sec.

### MRI data acquisition

Scanning was conducted on a 3T Siemens Trio scanner (Erlangen, Germany) with an eight-channel phase-arrayed coil at BNU Imaging Center for Brain Research, Beijing, China. Twenty-five 4 mm thick (20% skip) near axial slices were collected (in-plane resolution = 3×3 mm), oriented parallel to each subject's temporal cortex and covered the whole brain. T2*-weighted gradient-echo, echo-planar imaging procedures (EPI) were used (TR = 2 sec, TE = 32 ms, flip angle = 90°). In addition, MPRAGE, an inversion prepared gradient echo sequence (TR/TE/TI = 2.73s/3.44ms/1s, flip angle  = 7 deg, voxel size 1.1×1.1×1.9 mm), was used to acquire 3D structural images.

### Data analysis

Functional data were analyzed with the Freesurfer functional analysis stream (Cortechs Inc, Charlestown, MA) [41], [42], the fROI software (http://froi.sourceforge.net), and in-house Matlab code. After data preprocessing, including motion correction, intensity normalization, and spatial smoothing (Gaussian kernel, 5 mm full width at half maximum), voxel time courses for each individual subject were fitted by a general linear model (GLM). Each condition was modeled by a boxcar regressor matching its time course that was then convolved with a gamma function (delta = 2.25, tau = 1.25).

**Figure 3:**
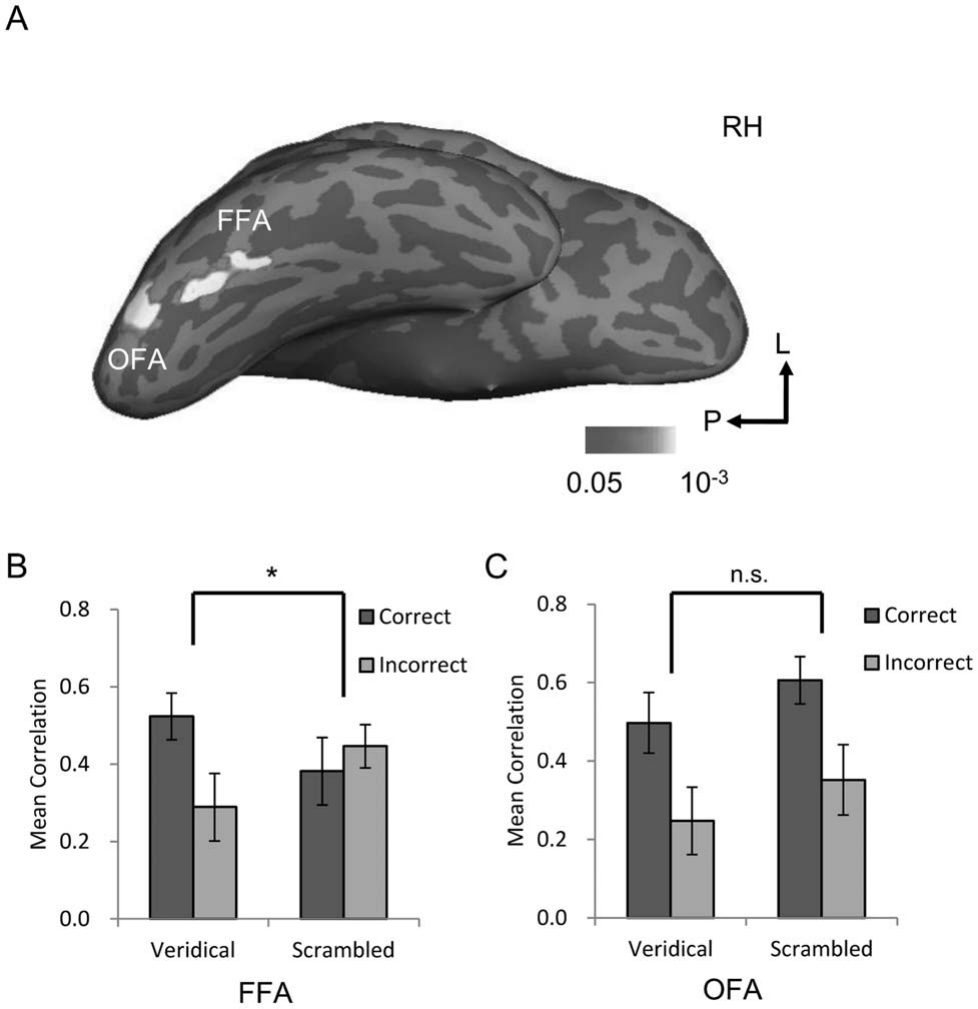
The behavioral relevance of the spatial patterns of neural activation in the face-selective regions. *A*) Face-selective regions of a typical participant. Face-selective regions, the FFA and OFA, from the fMRI localizer runs are shown on the right hemisphere (RH) of the inflated brain. Sulci are shown in dark gray and gyri in light gray. *B*) In the FFA, correlations of spatial patterns were higher for correct than incorrect trials only when face parts were presented in a veridical face configuration. * indicates that the two-way interaction of stimulus condition (Veridical versus Scrambled) by response type (Correct versus Incorrect) is significant. *C*) In the OFA, correlations of spatial patterns were higher for correct than incorrect trials, regardless of configural changes. Error bars indicate s.e.m. above and below the mean. *: *p*<0.05; n.s.: not significant.

For the localizer runs, we used a standard localizer method to identify regions of interest (ROI) separately for each hemisphere and for each participant (Table 1).Specifically, the FFA was defined as the set of contiguous voxels in the mid-fusiform gyrus that showed significantly higher responses to faces as compared with objects (*p*<0.01, uncorrected) (Fig. 3*A*).The relatively modest threshold used here was to obtain a decent number of voxels for MVPA. Another face-selective region, the occipital face area (OFA) [43], was defined in the same way but localized in the inferior occipital cortex (Fig. 3*A*). Two sub-regions of the lateral occipital complex (LOC) [44], [45], the posterior lateral occipital region (LO) and the anterior region in the posterior fusiform gyrus (pFs), were defined as those regions that responded more strongly to objects than scrambled objects. The functionalities of the OFA, LO, and pFs were examined to provide a contrast to that of the FFA. The FFA was successfully localized in all participants in the right hemisphere and in 11 out of 13 participants in the left, whereas the OFA was localized in 12 out of 13 in the right and only 9 out of 13 in the left. The LO and pFs were bilaterally localized in all participants. Because faces are processed more dominantly in the right hemisphere and because the ROIs in this study were localized more consistently in the right hemisphere, we chose to restrict our ROI-based analyses to regions in the right hemisphere (unless noted otherwise).

**Table 1:** Talairach coordinates of ROIs averaged across participants (Mean ± SD).

ROI	Hemisphere	Talairach coordinates			Voxel numbers	Number of Participants
		x	y	z		
FFA	Right	44±4	−54±7	−14±4	28±16	13
	Left	−42±5	−56±11	−14±5	18±9	11
OFA	Right	35±5	−80±7	−6±2	32±26	12
	Left	−37±3	−87±9	−8±3	30±30	9
LO	Right	42±5	−78±6	7±8	131±69	13
	Left	−42±4	−81±4	6±9	174±73	13
pFs	Right	35±5	−41±8	−18±5	25±16	13
	Left	−37±6	−46±6	−16±4	60±43	13

FFA: the fusiform face area; OFA: the occipital face area; LO: the lateral occipital cortex; pFs: the posterior fusiform gyrus; Number of Participants: the number of participants whose ROIs were successfully localized.

For the experimental runs, instead of pooling BOLD responses across voxels in the ROIs, MVPA was used to measure neural information encoded at a finer-scale. Specifically, we examined whether the spatial pattern of activation in the FFA, as well as the other pre-defined ROIs, was read out for behavioral face perception. That is, we compared the similarity of spatial patterns of activation in an ROI when participants correctly discriminated face pairs (correct trials) versus when they made incorrect responses (incorrect trials). Trials in which participants did not make a response (less than 0.1 % of total trials) were excluded from the analysis. For this analysis, the data were first preprocessed without spatial smoothing to maximize the sensitivity to any information present in the spatial pattern of activation. Then, we estimated the magnitude of the BOLD response with a gamma function (delta = 2.25, tau = 1.25) to each pair of faces as a compound trial for each voxel of each ROI. Finally, the ten experimental runs were split into five odd runs and five even runs, and the averaged spatial pattern was extracted from each half of the data (odd versus even runs) separately for each ROI, stimulus type (veridical versus scrambled), and behavioral response (correct versus incorrect). Within each ROI, we computed the correlation between the averaged spatial patterns on odd and even runs for veridical faces and for scrambled faces respectively; this analysis was performed separately on the data from trials in which the participant performed correctly versus incorrectly in the whole-part task. Before calculating the correlations, mean magnitude of activation across all conditions was subtracted from the magnitude of each condition in each voxel and in each half of the data. In addition to the MVPA, we also calculated the mean magnitude for each stimulus condition and for each response type by pooling BOLD responses across voxels.

## Results

### The absence of face configurations impairs discrimination of face parts

In the MRI scanner, face pairs, either both veridical or both scrambled (Fig. 1*A*), were presented sequentially (Fig. 2*A*). Participants performed a task to discriminate face parts between the faces in a pair. Behavioral data collected in the scanner showed that the participants were less accurate (*t*(12) = 8.87, *p*<0.001, Cohen's *d* = 2.53, veridical: 84.7 %, scrambled: 72.7 %) and slower (*t*(12) = 5.95, *p*<0.001, Cohen's *d* = 1.70) in discriminating face parts when they were placed in a non-face configuration (Scrambled) than in a veridical face configuration (Veridical) (Fig. 2B). Thus, faces are processed not on the basis of their individual parts, but rather on the basis of their overall shape, even when the participants were explicitly instructed to discriminate face parts. The influence of face configurations on the perception of face parts suggests a holistic face representation in the brain. Next, we examined whether the FFA is the neural substrate for this holistic representation.

### Faces are represented as integrated wholes in the FFA

Spatial patterns were extracted from each half of the data (odd versus even runs) separately for each stimulus condition (Veridical versus Scrambled faces) and for each response type (correct versus incorrect responses). Within an ROI, we calculated correlations between the spatial patterns in odd and even runs from trials in which participants performed correctly versus incorrectly in discriminating face parts either with or without face configurations.

A repeated-measures ANOVA of stimulus condition by response type on FFA responses revealed a significant two-way interaction (*F*(1,12)  = 5.09, *p* = 0.04, partial η^2^ = 0.30) (Fig. 3*B*). The main effects of stimulus condition (*F*(1,12) <1) and response type (*F*(1,12)  = 1.38, *p* = 0.26) were not significant. *Post-hoc* pair-wise t-tests found that when a veridical face configuration was present, spatial patterns of activation in the FFA were more similar among correct than incorrect trials (*t*(12)  = 3.22, *p* = 0.007, Cohen's *d* = 0.92). In contrast, when the configuration was scrambled, there was no effect of correct versus incorrect on the correlations between the spatial patterns (*t*(12) <1). We also matched the number of correct and incorrect trials between the two stimulus types by randomly selecting a subset of the data for a stimulus type that had more correct (or incorrect) trials. With the matched number of trials, we found a similar result – a significantly higher correlation in the FFA for correct compared with incorrect trials was observed when the face configuration was present (*t*(12)  = 3.94, *p* = 0.002, Cohen's *d* = 1.23), not when the face configuration was absent (*t*<1) (Fig. S1). In addition, in the left FFA neither the interaction (*F*<1) nor the difference between the correct and incorrect trials (both *ts* <1) was found, consistent with previous findings that the holistic representation of faces is found only in the right FFA [46]. Finally, with the traditional GLM analysis using mean magnitudes of BOLD responses, we failed to find an interaction of stimulus condition by response type (*F*<1) (Fig. S2). This may be accounted for by the fact that FFA response to scrambled faces was almost as strong as that to veridical faces (Fig. S3), making the effect of holistic processing too weak to be detected based on mean BOLD magnitude alone.

The interdependence of the featural and configural information in the FFA suggests that faces are represented not on the basis of their individual parts but in terms of the whole that emerges from the parts, consistent with the Holistic model (Fig. 1*C*). However, an alternative interpretation may account for this finding: veridical faces were more familiar to the participants than scrambled faces. We ruled out this alternative by examining the behavioral relevance of the spatial pattern in the OFA. A two-way ANOVA revealed a main effect of response type (*F(1,11)*  = 21.14, *p*<0.001, partial η^2^ = 0.66) (Fig. 3*C*), indicating that the spatial patterns of activation in the OFA were more similar among correct than incorrect trials. Importantly, there was no interaction of stimulus condition by response type (*F(1,11)* <1), showing that the OFA has no preference for stimuli with veridical face configurations (*t*(11)  = 1.94, *p* = 0.08, Cohen's *d* = 0.56) over stimuli without such configurations (*t*(11)  = 3.64, *p* = 0.004, Cohen's *d* = 1.13). Therefore, the OFA is apparently engaged in the parts-based representation of faces (Parts-based Model, Fig. 1*D*), which is qualitatively different from the representation in the FFA. The functional division of labor between the OFA and FFA is further supported by a significant interaction of cortical region (OFA versus FFA) by response type (correct versus incorrect) when face configurations were absent (*F*(1,11)  = 6.13, *p* = 0.03, partial η^2^ = 0.36), confirming that the configural information is necessary for the featural information encoded in the FFA to be read out for behavioral face perception. The three-way interaction among cortical region (FFA versus OFA), response type (correct versus incorrect), and stimulus type (Veridical versus Scrambled) did not reach significance (*F*(1, 11)  = 2.73, *p* = 0.13).

### The hierarchical representation of faces is not found in the object system

The functional division of labor between the OFA and FFA suggests that the FFA and the OFA may comprise a hierarchical network for face processing, with the FFA inheriting the parts-based representation of faces in the OFA, and then integrating this information with their spatial relations for the holistic representation of the faces. Here we examined the specificity of the hierarchy of face representation by investigating two regions in the object processing hierarchy, the LO and the pFs, that are located adjacent to the OFA and the FFA, respectively.

**Figure 4:**
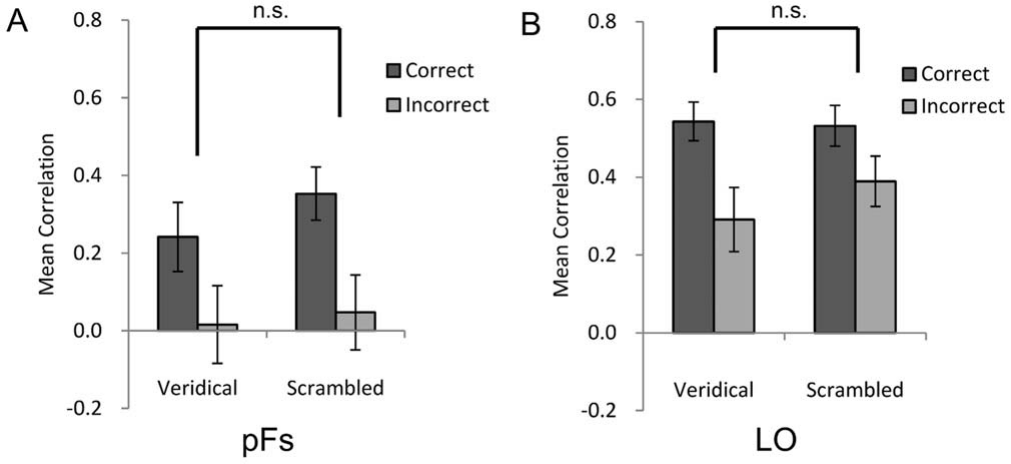
The behavioral relevance of the object-selective regions. Both the pFs (*A*) and LO (*B*) were involved in the parts-based representation of faces, as correlations of spatial patterns of activation in both regions were higher for correct trials than incorrect trials, regardless of configural changes. Error bars indicate s.e.m. above and below the mean.

The spatial patterns in both the LO and pFs were more similar among correct trials than incorrect trials, regardless of whether face configurations were present (LO: *t*(12)  = 3.35, *p* = 0.006, Cohen's *d* = 1.02; pFs: *t*(12)  = 1.86, *p* = 0.09, Cohen's *d* = 0.50) or absent (LO: *t*(12)  = 2.35, *p* = 0.04, Cohen's *d* = 0.49; pFs: *t*(12)  = 3.25, *p* = 0.007, Cohen's *d* = 0.80) (Fig. 4). Importantly, there was no significant interaction between the LO and the pFs by response type when face configurations were absent (*F*(1,12)  = 2.88, *p* = 0.12), further confirming that faces are represented non-interactively in both the LO and the pFs. Thus, for the ROIs tested, the holistic representation of faces is specifically carried out in the FFA, whereas the parts-based representation of faces is not.

## Discussion

In this study, we asked whether the featural information of faces is encoded independently of or combined with the configural information of faces in the FFA, by correlating spatial patterns of activation in FFA with a measure of behavioral face perception. In the experiment, participants were instructed to discriminate face parts that were placed either in a veridical or in a scrambled face configuration. We found that the absence of the first-order face configuration impaired discrimination of face parts, showing the importance of overall structure in representing faces. More importantly, only when the face parts were placed in a veridical face configuration was the featural information in the FFA related to behavioral performance. Therefore, face parts were not independently and non-interactively represented in the FFA, suggesting that the FFA is only engaged in the holistic representation of faces.

Our study applied several novel methods to assess the nature of the representations in the FFA. First, unlike previous studies where FFA responses to face parts were compared with visually similar stimuli (e.g., black ovals) [5], in this study, spatial patterns of activation in the FFA were pooled by behavioral response (i.e., correct versus incorrect responses), while stimuli were kept constant. This approach largely avoided confounding factors such as differences in low-level properties among stimuli. Second, in previous studies, face parts were usually presented in a veridical face configuration [9], [23]. However, this paradigm may be problematic, because the presence of veridical face configurations, although irrelevant to the task, automatically interferes with the processing of face parts [26]. In this study, we re-arranged face parts in a non-face configuration, and therefore achieved a relatively pure measure of the featural information present. Finally, instead of simply comparing neural activation across stimuli or tasks, we examined whether spatial patterns of the activation in the FFA were read out for behavioral face perception. The analysis of the behavioral relevance of neural activation helps differentiate activation that represents faces from activation that is simply epiphenomenal [38], [47].

Our finding dovetails with previous studies showing that the FFA is involved in the holistic representation of faces [15], [16], [46]. Our study further demonstrates that the behavioral relevance of the featural information depended on the presence of veridical face configurations, ruling out the possibility that the FFA is also involved in the parts-based representation of faces. That is, the FFA is engaged in holistic representation of faces, where the face is represented as an integrated whole, rather than as a collection of independent components. However, it is less clear how the presence of the first-order face configuration facilitates the representation of faces in the FFA. One possibility is that face configurations may help coordinate activities of neurons in the FFA that are sensitive to different face parts so that the neurons can work in a cohort for representing faces, possibly through population coding [48].

In contrast to the results we observed in the FFA, the OFA is engaged in the parts-based representation of faces. Spatial patterns of activation in the OFA were more similar among correct than incorrect trials, regardless of configural changes. This result fits with a TMS study where TMS stimulation of the OFA selectively disrupted participants' ability to discriminate face parts, not their ability to discriminate face configurations [5], [15], [49]–[52]. The parts-based representation found in the OFA is also consistent with the observation that the participants performed the task above chance-level when face parts were placed in a non-face configuration. However, the neural information in the OFA alone is not sufficient for optimal behavioral face perception. A higher performance in discriminating face parts with the presence of face configurations may be explained by the contribution of the neural information encoded in the FFA. Such information was lost in behavioral performance when face configurations were scrambled.

The dissociation between the holistic representation and parts-based representation of faces in the brain suggests a functional division of labor between the OFA and FFA. One possible explanation from a widely-held “local-to-global” view [53]–[55] is that the OFA and the FFA may process faces at difference scales, with local properties of faces extracted in the OFA (i.e., face parts) and then more global properties extracted in the FFA for further holistic representation. However, our study suggests otherwise, as the LO and pFs, two regions in the object processing hierarchy along the ventral pathway, were only involved in the parts-based representation of faces [8], [9], [54]. Therefore, the functional division of labor observed between the OFA and FFA may arise from a qualitative difference at different stages of representation for faces, not merely from the quantitative increase in sensitivity to the global properties of objects along the ventral pathway [52], [56]. In addition to the differences in function between the FFA and OFA, typical face processing requires collaborative efforts from both regions [57]. For example, the FFA can be preserved in an individual with a lesion to the OFA who, as a result, suffers from prosopagnosia [51], [58]. Future studies on the nature of the dynamic links between the FFA and other cortical regions such as the OFA may help answer whether and how interactions with other regions modulate the computations conducted in the FFA.

## Supporting Information

Figure S1 **The behavioral relevance of spatial patterns in the FFA with matched number of trials.** The numbers of correct and incorrect trials between the two stimulus types were matched by randomly selecting a subset of the data for stimulus type that had more correct (or incorrect) trials. Error bars indicate s.e.m. above and below the mean.
(PDF)

Figure S2 **Mean magnitudes of BOLD responses in the FFA.** The magnitude was acquired by the traditional GLM analysis. A two-way ANOVA revealed no interaction of stimulus condition (Veridical versus Scrambled) by response type (correct versus incorrect) in the FFA (*F* <1). *Post hoc* pair-wise t-tests revealed no significant difference between the correct and incorrect trials in either veridical or scrambled condition (*ts* <1).
(PDF)

Figure S3 **Mean magnitude of BOLD responses in the FFA to stimuli.** A) FFA responses to faces with or without veridical face configurations. The FFA showed a strong response to the scrambled faces, which was about 93% of the activation level to the veridical faces. B) FFA responses to faces and objects in the localizer runs. Note that the data shown here were not from an independent data set (i.e., the same set used to define the ROIs). Previous studies based on independent data sets show that FFA response to faces is at least two times higher than that to objects [e.g., 1].
(PDF)

## References

[1] Kanwisher N, McDermott J, Chun MM (1997). The fusiform face area: a module in human extrastriate cortex specialized for face perception.. J Neurosci.

[2] Barton JJ, Press DZ, Keenan JP, O'Connor M (2002). Lesions of the fusiform face area impair perception of facial configuration in prosopagnosia.. Neurology.

[3] Harris A, Aguirre GK (2008). The representation of parts and wholes in face-selective cortex.. J Cogn Neurosci.

[4] Harris A, Aguirre GK (2010). Neural tuning for face wholes and parts in human fusiform gyrus revealed by FMRI adaptation.. J Neurophysiol.

[5] Liu J, Harris A, Kanwisher N (2010). Perception of face parts and face configurations: an FMRI study.. J Cogn Neurosci.

[6] Maurer D, Ocraven K, Legrand R, Mondloch C, Springer M (2007). Neural correlates of processing facial identity based on features versus their spacing.. Neuropsychologia.

[7] Rhodes G, Michie PT, Hughes ME, Byatt G (2009). The fusiform face area and occipital face area show sensitivity to spatial relations in faces.. European Journal of Neuroscience.

[8] Rotshtein P, Geng JJ, Driver J, Dolan RJ (2007). Role of features and second-order spatial relations in face discrimination, face recognition, and individual face skills: behavioral and functional magnetic resonance imaging data.. J Cogn Neurosci.

[9] Yovel G, Kanwisher N (2004). Face PerceptionDomain Specific, Not Process Specific.. Neuron.

[10] Maurer D, Grand RL, Mondloch CJ (2002). The many faces of configural processing.. Trends Cogn Sci.

[11] McKone E, Martini P, Nakayama K (2001). Categorical perception of face identity in noise isolates configural processing.. J Exp Psychol Hum Percept Perform.

[12] Sergent J (1984). An investigation into component and configural processes underlying face perception.. British Journal of Psychology.

[13] Tanaka JW, Farah MJ (1993). Parts and wholes in face recognition.. Q J Exp Psychol A.

[14] Young AW, Hellawell D, Hay DC (1987). Configurational information in face perception.. Perception.

[15] Schiltz C (2010). Holistic perception of individual faces in the right middle fusiform gyrus as evidenced by the composite face illusion.. Journal of Vision.

[16] Schiltz C, Rossion B (2006). Faces are represented holistically in the human occipito-temporal cortex.. NeuroImage.

[17] Cabeza R, Kato T (2000). Features are also important: contributions of featural and configural processing to face recognition.. Psychol Sci.

[18] Diamond R, Carey S (1986). Why faces are and are not special: an effect of expertise.. J Exp Psychol Gen.

[19] Rhodes G (1988). Looking at faces: first-order and second-order features as determinants of facial appearance.. Perception.

[20] Barton JJ, Keenan JP, Bass T (2001). Discrimination of spatial relations and features in faces: Effects of inversion and viewing duration.. Br J Psychol 92 Part.

[21] Freire A, Lee K, Symons LA (2000). The face-inversion effect as a deficit in the encoding of configural information: direct evidence.. Perception.

[22] Leder H, Bruce V (2000). When inverted faces are recognized: the role of configural information in face recognition.. Q J Exp Psychol A.

[23] Riesenhuber M, Jarudi I, Gilad S, Sinha P (2004). Face processing in humans is compatible with a simple shape-based model of vision.. Proceedings of the Royal Society B: Biological Sciences.

[24] Yovel G, Kanwisher N (2008). The representations of spacing and part-based information are associated for upright faces but dissociated for objects: evidence from individual differences.. Psychon Bull Rev.

[25] Le Grand R, Mondloch CJ, Maurer D, Brent HP (2004). Impairment in holistic face processing following early visual deprivation.. Psychol Sci.

[26] Zhu Q, Li X, Chow K, Liu J (2009). The part task of the part-spacing paradigm is not a pure measurement of part-based information of faces.. PLoS One.

[27] Farah MJ, Wilson KD, Drain M, Tanaka JN (1998). What is “special” about face perception?. Psychol Rev.

[28] Tanaka JW, Sengco JA (1997). Features and their configuration in face recognition.. Mem Cognit.

[29] Cox DD, Savoy RL (2003). Functional magnetic resonance imaging (fMRI) “brain reading”: detecting and classifying distributed patterns of fMRI activity in human visual cortex.. NeuroImage.

[30] Haxby JV, Gobbini MI, Furey ML, Ishai A, Schouten JL (2001). Distributed and overlapping representations of faces and objects in ventral temporal cortex.. Science.

[31] Haynes JD, Rees G (2006). Decoding mental states from brain activity in humans.. Nat Rev Neurosci.

[32] Norman KA, Polyn SM, Detre GJ, Haxby JV (2006). Beyond mind-reading: multi-voxel pattern analysis of fMRI data.. Trends Cogn Sci.

[33] Peelen MV, Downing PE (2007). Using multi-voxel pattern analysis of fMRI data to interpret overlapping functional activations.. Trends Cogn Sci.

[34] Schurger A, Pereira F, Treisman A, Cohen JD (2010). Reproducibility distinguishes conscious from nonconscious neural representations.. Science.

[35] Xue G, Dong Q, Chen C, Lu Z, Mumford JA (2010). Greater neural pattern similarity across repetitions is associated with better memory.. Science.

[36] Li S, Mayhew SD, Kourtzi Z (2009). Learning Shapes the Representation of Behavioral Choice in the Human Brain.. Neuron.

[37] Raizada RDS, Tsao FM, Liu HM, Kuhl PK (2009). Quantifying the Adequacy of Neural Representations for a Cross-Language Phonetic Discrimination Task: Prediction of Individual Differences.. Cerebral Cortex.

[38] Williams MA, Dang S, Kanwisher NG (2007). Only some spatial patterns of fMRI response are read out in task performance.. Nature Neuroscience.

[39] Hosie JA, Ellis HD, Haig ND (1988). The effect of feature displacement on the perception of well-known faces.. Perception.

[40] Rhodes G, Brake S, Atkinson AP (1993). What's lost in inverted faces?. Cognition.

[41] Dale AM, Fischl B, Sereno MI (1999). Cortical surface-based analysis. I. Segmentation and surface reconstruction.. NeuroImage.

[42] Fischl B, Sereno MI, Dale AM (1999). Cortical surface-based analysis. II: Inflation, flattening, and a surface-based coordinate system.. NeuroImage.

[43] Gauthier I, Tarr MJ, Moylan J, Skudlarski P, Gore JC (2000). The fusiform “face area” is part of a network that processes faces at the individual level.. J Cogn Neurosci.

[44] Grill-Spector K, Kushnir T, Hendler T, Malach R (2000). The dynamics of object-selective activation correlate with recognition performance in humans.. Nat Neurosci.

[45] Kourtzi Z, Kanwisher N (2001). Representation of perceived object shape by the human lateral occipital complex.. Science.

[46] Rossion B, Dricot L, Devolder A, Bodart JM, Crommelinck M (2000). Hemispheric asymmetries for whole-based and part-based face processing in the human fusiform gyrus.. J Cogn Neurosci.

[47] Grill-Spector K, Knouf N, Kanwisher N (2004). The fusiform face area subserves face perception, not generic within-category identification.. Nature Neuroscience.

[48] Hirabayashi T, Miyashita Y (2005). Dynamically modulated spike correlation in monkey inferior temporal cortex depending on the feature configuration within a whole object.. J Neurosci.

[49] Pitcher D, Walsh V, Yovel G, Duchaine B (2007). TMS Evidence for the Involvement of the Right Occipital Face Area in Early Face Processing.. Current Biology.

[50] McCarthy G, Puce A, Belger A, Allison T (1999). Electrophysiological studies of human face perception. II: Response properties of face-specific potentials generated in occipitotemporal cortex.. Cereb Cortex.

[51] Rossion B, Caldara R, Seghier M, Schuller AM, Lazeyras F (2003). A network of occipito-temporal face-sensitive areas besides the right middle fusiform gyrus is necessary for normal face processing.. Brain.

[52] Rotshtein P, Henson RN, Treves A, Driver J, Dolan RJ (2005). Morphing Marilyn into Maggie dissociates physical and identity face representations in the brain.. Nat Neurosci.

[53] Barlow HB (1972). Single units and sensation: a neuron doctrine for perceptual psychology?. Perception.

[54] Lerner Y, Hendler T, Ben-Bashat D, Harel M, Malach R (2001). A hierarchical axis of object processing stages in the human visual cortex.. Cereb Cortex.

[55] Tanaka K (1996). Inferotemporal cortex and object vision.. Annu Rev Neurosci.

[56] Yovel G, Kanwisher N (2005). The Neural Basis of the Behavioral Face-Inversion Effect.. Current Biology.

[57] Zhu Q, Zhang J, Luo YL, Dilks DD, Liu J (2011). Resting-state neural activity across face-selective cortical regions is behaviorally relevant.. J Neurosci.

[58] Steeves JK, Culham JC, Duchaine BC, Pratesi CC, Valyear KF (2006). The fusiform face area is not sufficient for face recognition: evidence from a patient with dense prosopagnosia and no occipital face area.. Neuropsychologia.

