# Supplementary material for: The Fusiform Face Area Is Engaged in Holistic, Not Parts-Based, Representation of Faces
Source: PLoS One. 2012 Jul 6;7(7):e40390. doi: 10.1371/journal.pone.0040390 (PMC3391267; doi:10.1371/journal.pone.0040390)
Supplement: Figure S1 — The behavioral relevance of spatial patterns in the FFA with matched number of trials. The numbers of correct and incorrect trials between the two stimulus types were matched by randomly selecting a subset of the data for stimulus type that had more correct (or incorrect) trials. Error bars indicate s.e.m. above and below the mean. (PDF) [file pone.0040390.s001.pdf]

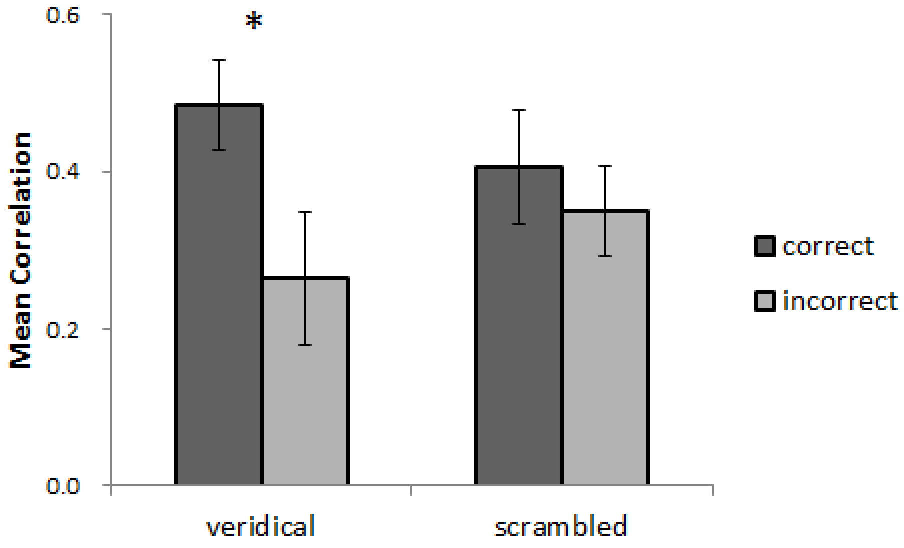

Figure S1. The behavioral relevance of spatial patterns in the FFA with matched number of trials. The numbers of correct and incorrect trials between the two stimulus types were matched by randomly selecting a subset of the data for stimulus type that had more correct (or incorrect) trials. Error bars indicate s.e.m. above and below the mean.
