# Supplementary material for: The Fusiform Face Area Is Engaged in Holistic, Not Parts-Based, Representation of Faces
Source: PLoS One. 2012 Jul 6;7(7):e40390. doi: 10.1371/journal.pone.0040390 (PMC3391267; doi:10.1371/journal.pone.0040390)
Supplement: Figure S2 — Mean magnitudes of BOLD responses in the FFA. The magnitude was acquired by the traditional GLM analysis. A two-way ANOVA revealed no interaction of stimulus condition (Veridical versus Scrambled) by response type (correct versus incorrect) in the FFA (F <1). Post hoc pair-wise t-tests revealed no significant difference between the correct and incorrect trials in either veridical or scrambled condition (ts <1). (PDF) [file pone.0040390.s002.pdf]

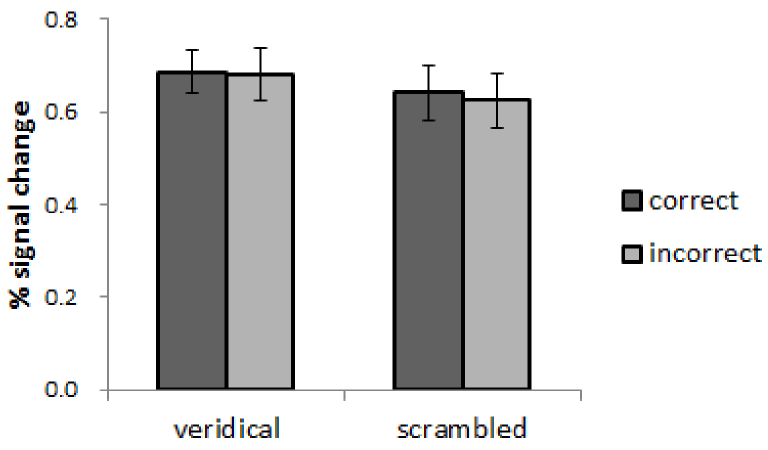

Figure S2. Mean magnitudes of BOLD responses in the FFA. The magnitude was acquired by the traditional GLM analysis. A two-way ANOVA revealed no interaction of stimulus condition (Veridical versus Scrambled) by response type (correct versus incorrect) in the FFA ($F < 1$). *Post hoc* pair-wise t-tests revealed no significant difference between the correct and incorrect trials in either veridical or scrambled condition ($ts < 1$).
