# Supplementary material for: The Fusiform Face Area Is Engaged in Holistic, Not Parts-Based, Representation of Faces
Source: PLoS One. 2012 Jul 6;7(7):e40390. doi: 10.1371/journal.pone.0040390 (PMC3391267; doi:10.1371/journal.pone.0040390)
Supplement: Figure S3 — Mean magnitude of BOLD responses in the FFA to stimuli. A) FFA responses to faces with or without veridical face configurations. The FFA showed a strong response to the scrambled faces, which was about 93% of the activation level to the veridical faces. B) FFA responses to faces and objects in the localizer runs. Note that the data shown here were not from an independent data set (i.e., the same set used to define the ROIs). Previous studies based on independent data sets show that FFA response to faces is at least two times higher than that to objects [e.g., 1]. (PDF) [file pone.0040390.s003.pdf]

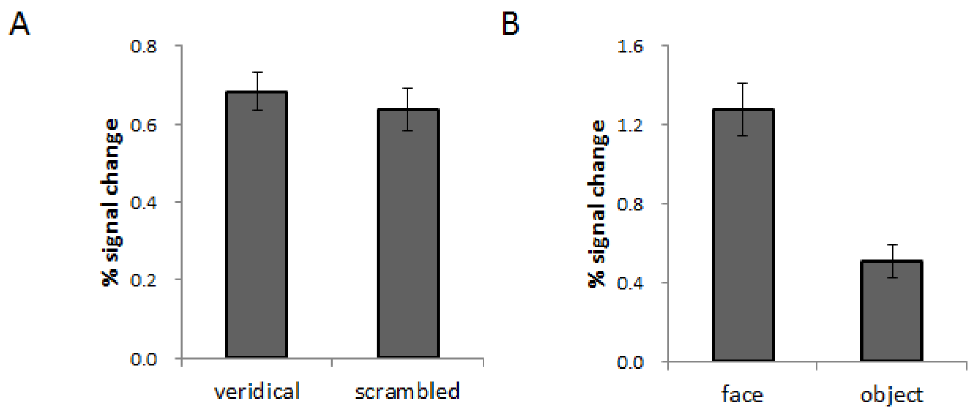

Figure S3: Mean magnitude of BOLD responses in the FFA to stimuli. A) FFA responses to faces with or without veridical face configurations. The FFA showed a strong response to the scrambled faces, which was about 93% of the activation level to the veridical faces. B) FFA responses to faces and objects in the localizer runs. Note that the data shown here were not from an independent data set (i.e., the same set used to define the ROIs). Previous studies based on independent data sets show that FFA response to faces is at least two times higher than that to objects [e.g., 1].
